# Epidemiological characteristics and gender-specific differences of obstructive sleep apnea in a Chinese hypertensive population: a cross-sectional study

**DOI:** 10.1186/s12872-016-0447-4

**Published:** 2017-01-05

**Authors:** Anping Cai, Yingling Zhou, Jiawei Zhang, Qi Zhong, Rui Wang, Ling Wang

**Affiliations:** Department of Cardiology, Guangdong Cardiovascular Institute, Guangdong Provincial Key Laboratory of Coronary Heart Disease Prevention, Guangdong General Hospital, Guangdong Academy of Medical Sciences, 106 Zhongshan Road 2, Guangzhou, 510080 China

**Keywords:** Obstructive sleep apnea, Hypertension, Epidemiology

## Abstract

**Background:**

Obstructive sleep apnea (OSA) is associated with an increase in the prevalence and incidence of hypertension and cardiovascular diseases. Data about epidemiological characteristics of OSA in Chinese hypertensive populations is limited.

**Methods:**

Hypertensive subjects without a prior diagnosis of OSA were recruited, and the apnea-hyponea index (AHI) was assessed by polysomnography. Comparisons were performed between subjects without OSA and with different degrees of OSA. Gender-specific differences in epidemiological characteristics of OSA were also analyzed. Univariate and multivariate regression analyses were conducted to evaluate the associations between OSA and other variables.

**Results:**

A total of 971 hypertensive subjects were enrolled and 685 (70.5%) were diagnosed with OSA. Compared to those without OSA, subjects with OSA were more likely male (78.4% versus 71.7%, *P* = 0.016) and at higher cardiovascular risk in subjects with moderate-severe OSA. Among the 685 OSA subjects, 79.4% (537 cases) were males. Gender-specific differences in epidemiological characteristics of OSA were observed. Multivariate regression analyses revealed that after adjusting for covariates, only body mass index positively correlated with OSA in males (odds ratio (OR): 1.064, 95% confidence interval (CI): 1.008–1.123, *P* = 0.024). In female subjects, after adjusting for covariates, only age positively correlated with OSA (OR: 1.071, 95% CI: 1.029–1.116, *P* = 0.001).

**Conclusion:**

In summary, in a Chinese hypertensive population, OSA prevalence is strikingly high. Hypertensive subjects with the most severe OSA are at greater cardiovascular risk. There are significant differences in epidemiological characteristics of OSA between male and female.

## Background

Obstructive sleep apnea (OSA) is a major public health problem worldwide, and is associated with an increase in the prevalence and incidence of arterial hypertension and cardiovascular diseases (CVD) [1, 2]. Repetitive obstructive apneas appear to initiate a variety of pathophysiological effects which subsequently act to promote and accelerate the development of CVD [3–5]. A number of markers associated with OSA have been identified, and male gender is considered to be a major independent risk factor [6]. Data from population-based studies reveal that compared to females, the prevalence of OSA in males is 1.5 to 3 times greater, and in undiagnosed OSA patients, the ratio of males to females was nearly 2:1 [7–9]. These data strongly suggest OSA has a male predilection. Nevertheless, these findings may also indirectly reflect a relative lack of awareness and consequent under-evaluation of OSA in females [9]. Furthermore, it has been reported that female OSA patients may have greater improvement in cardiovascular function than their male counterparts with appropriate treatment [10]. These data collectively indicate the clinical importance of promoting awareness of OSA in females. They further underscore that a better understanding of the differences in epidemiological characteristics between male and female OSA populations is also a top priority.

In recent decades, the prevalence of arterial hypertension, a frequent co-morbidity of OSA, has increased, likely as a consequence of the increased prevalence of OSA. However, thus far, evidence regarding epidemiological characteristics of OSA in Chinese populations is limited. Moreover, whether there are gender-specific differences in clinical features of OSA is also unknown in the Chinese populations. We hypothesized that the prevalence of OSA might be positively related to increased prevalence of hypertension and gender-specific differences of OSA characteristics might also exist. In order to demonstrate our hypotheses, we therefore conducted a cross-sectional study to investigate the prevalence and epidemiological characteristics of OSA in randomly selected hypertensive patients in our inpatient department. We further investigated the differences in epidemiological characteristics of OSA between male and female hypertensive subjects. Hopefully, our present research will provide basis for the cohort and intervention studies in the Chinese OSA populations in the future.

## Methods

### Studied participants

This study was approved by the clinical research ethics committee of Guangdong General Hospital (No. GDREC 2015373H), and informed consent was obtained before participants were enrolled. Included criteria were as follows: previous diagnosis of essential hypertension and had not been previously diagnosed with OSA. Excluded criteria were as follows: pre-hypertensive stage or documented secondary hypertension, or had a history of OSA or treatment with continuous positive airway pressure or devices. Polysomnography (PSG) was performed to evaluate the apnea-hypopnea index (AHI). All participants had been informed the detailed procedures of PSG. Participants were allowed to have their regular sleep-wake rhythm, while substances such as alcohol or sleeping medicines were not allowed to take during PSG performance. In brief, if the patients had airflow complete blockage for more than 10 s or > 50% reduction in respiratory airflow accompanying > 3% reduction in SaO_2_ for more than 10 s, the apnea or hypopnea events would be recorded. In brief, on the basis of AHI as defined by the total number of apneas and hypopneas per sleep hour based on current AASM guidelines. Participants with AHI of 5–14 were defined as mild OSA, 15–29 were moderate and 30 or more were severe. Less than 5 were considered without OSA. All measurements were assessed by using PHILIPS RESPIRONICS Alice PDx and the validity and reliability of this device and measures had been broadly demonstrated our previous study [11].

### Clinical and laboratory data collection

Clinical data including demographics, anthropometrics, previous medical history and medicine usage were recorded on a clinical report form by two investigators and underwent re-checks by two additional investigators. Fasting venous blood was sampled for measurements of lipid profiles, fasting plasma glucose (FPG) and glycated hemoglobin (HbA1c). Echocardiography was used to evaluate thickness of the inter-ventricular septum (IVS) and left ventricular posterior wall (LVPW).

### Study design

This was a cross-sectional single-center study. Initially, all participants were separated into those with OSA and those without OSA, and comparisons were performed. Thereafter, all participants were divided into 3 groups, namely without OSA, mild OSA, and moderate-severe OSA groups, and clinical characteristics of these three groups were evaluated and compared. Participants with OSA were then separated into male and female groups, and between-group differences were analyzed. Last, univariate and multivariate regression analyses were conducted to analyze and compare the associations between OSA and other clinical variables in the male and female populations, respectively.

### Statistical analysis

Standard descriptive statistics were in the analysis. Continuous variables were described using mean and SD. Categorical variables were described by the percentages of categories. The statistical significance of differences is analyzed using one-way ANOVA or Mann–Whitney *U* test for continuous variables and the chi-square or Fisher exact test for categorical variables. Univariate and multivariate regression analyses were used to evaluate the association between OSA and clinical and laboratory variables. Statistical analyze were computed using SPSS 18.0 (SPSS Inc, Chicago, IL). All statistical tests were two-sided and considered statistically significant when *P* < 0.05.

## Results

### Patient characteristics

A total of 971 hypertensive subjects were enrolled, 76.4% of whom were male. Among these 971 subjects, 70% were diagnosed with OSA. Comparisons were performed between subjects with and without OSA. Compared to subjects without OSA, those with OSA were older, more likely males and had higher body mass index (BMI), IVS, LVPW and HbA1c (*P* < 0.05 for all comparisons) (Table 1). Notably, compared to those without OSA, subjects with OSA appeared to be at higher cardiovascular risk as reflected by higher prevalence of proteinuria, diabetes mellitus, dyslipidemia, aneurysm, coronary heart disease (CHD), cerebrovascular disease, heart failure, atrial fibrillation and hypertrophic cardiomyopathy (HCM), though these between-group differences did not achieve statistical significance. With respect to medications, only statin and beta-blocker usage were significantly higher in the OSA group (*P* < 0.05); no significant differences in other medications were observed.

**Table 1:** Patient characteristics

Variables	Without OSA	With OSA	*P* value
N (%)	286 (29.5)	685 (70.5)	
Age (years)	56.5 ± 13.3	59.3 ± 11.7	0.001
Male (%)	71.7	78.4	0.016
BMI (Kg/m^2^)	24.6 ± 3.6	25.8 ± 4.1	<0.001
SBP (mm Hg)	137.3 ± 20.4	137.4 ± 21.5	0.975
DBP (mm Hg)	79.3 ± 13.6	79.4 ± 14.3	0.926
AHI	1.7 ± 1.4	22.0 ± 16.7	0.001
IVS (mm)	10.7 ± 1.8	11.4 ± 2.3	<0.001
LVPW (mm)	10.2 ± 1.8	10.5 ± 1.8	0.007
HbA1c (%)	6.1 ± 1.1	6.3 ± 1.2	0.029
Creatinine (μmol/L)	91.6 ± 48.0	99.0 ± 65.9	0.098
BUN (mmol/L)	5.5 ± 3.2	5.8 ± 2.8	0.162
FPG (mmol/L)	6.2 ± 2.6	6.5 ± 3.0	0.130
Triglyceride (mmol/L)	1.9 ± 0.3	1.8 ± 0.1	0.582
Total cholesterol (mmol/L)	4.5 ± 1.3	4.5 ± 1.2	0.643
LDL-C (mmol/L)	2.7 ± 1.0	2.7 ± 1.0	0.939
HDL-C (mmol/L)	1.0 ± 0.3	1.0 ± 0.2	0.961
Proteinuria (%)	17.1	20.4	0.183
Diabetes mellitus (%)	19.6	24.4	0.061
Dyslipidemia (%)	10.5	14.6	0.052
Aortic dissection (%)	20.3	19.9	0.472
Aneurysm (%)	4.5	5.4	0.354
CHD (%)	37.4	41.9	0.110
Cerebrovascular disease (%)	6.6	9.9	0.063
Heart failure (%)	19.2	21.9	0.200
Atrial fibrillation (%)	8.4	9.1	0.424
HCM (%)	1.7	3.4	0.121
Anti-platelet (%)	49.0	54.3	0.073
Statins (%)	59.1	66.9	0.013
ACEI (%)	22.0	22.0	0.534
ARB (%)	49.7	46.0	0.165
Beta-blocker (%)	59.4	66.4	0.023
Alpha-blocker (%)	6.6	6.4	0.500
CCB (%)	55.2	57.1	0.324
Diuretic (%)	16.1	18.7	0.192
Warfarin (%)	6.6	7.2	0.448

Denote: *BUN* blood uria nitrogen, *LDL-C* low-density lipoprotein cholesterol, *HDL-C* high-density lipoprotein cholesterol, *CHD* coronary heart disease, *HCM* hypertrophy cardiomyopahty, *ACEI* angiotensin converting enzyme inhibitor, *ARB* angiotensin receptor blocker, *CCB* calcium channel blocker

### Comparisons between OSA groups

Epidemiological characteristics of subjects with different degrees of OSA were compared. As shown in Table 2, compared to the mild OSA group, subjects in the moderate-severe OSA group were more likely males, had higher values of BMI, IVS, LVPW and creatinine, and significantly higher prevalence of proteinuria, diabetes mellitus and heart failure (*P* < 0.05 for all comparisons). In addition, dyslipidemia, aneurysm, CHD and HCM tended to be more frequent in the moderate-severe OSA group, although without statistical significance, indicating a relation between OSA severity and cardiovascular risk.

**Table 2:** Comparisons between different OSA groups

Variables	Without OSA	Mild	Moderate-severe	*P* value
N (%)	286 (29.5)	318 (32.7)	367 (37.8)	
Age (years)	56.5 ± 13.3	59.9 ± 11.4	58.8 ± 11.9	0.002
Male (%)	71.7	73.6	82.6	<0.001
BMI (Kg/m^2^)	24.6 ± 3.6	24.8 ± 3.4	26.6 ± 4.4	<0.001
SBP (mm Hg)	137.3 ± 20.4	137.7 ± 21.5	137.1 ± 21.5	0.934
DBP (mm Hg)	79.3 ± 13.6	79.3 ± 14.8	79.4 ± 14.0	0.985
AHI	1.7 ± 1.4	9.2 ± 2.7	33.1 ± 15.7	<0.001
IVS (mm)	10.7 ± 1.8	11.2 ± 2.6	11.5 ± 2.1	<0.001
LVPW (mm)	10.2 ± 1.8	10.3 ± 1.7	10.7 ± 1.9	0.001
HbA1c (%)	6.1 ± 1.1	6.2 ± 1.1	6.2 ± 1.2	0.838
Creatinine (μmol/L)	91.6 ± 48.0	92.7 ± 45.3	104.5 ± 79.2	0.012
BUN (mmol/L)	5.5 ± 3.2	5.7 ± 2.9	5.9 ± 2.8	0.290
FPG (mmol/L)	6.2 ± 2.6	6.3 ± 2.5	6.6 ± 3.4	0.097
Triglyceride (mmol/L)	1.9 ± 0.3	1.7 ± 0.1	1.9 ± 0.1	0.665
Total cholesterol (mmol/L)	4.5 ± 1.3	4.5 ± 1.3	4.6 ± 1.2	0.442
LDL-C (mmol/L)	2.7 ± 1.0	2.7 ± 1.0	2.7 ± 1.0	0.938
HDL-C (mmol/L)	1.0 ± 0.3	1.0 ± 0.2	1.0 ± 0.2	0.892
Proteinuria (%)	17.1	18.5	22.0	0.027
Diabetes mellitus (%)	19.6	23.0	25.6	0.038
Dyslipidemia (%)	10.5	14.2	15.0	0.057
Aortic dissection (%)	20.3	20.8	19.1	0.358
Aneurysm (%)	4.5	4.7	6.0	0.221
CHD (%)	37.4	39.9	43.6	0.058
Cerebrovascular disease (%)	6.6	11.0	9.0	0.195
Heart failure (%)	19.2	17.6	25.6	0.019
Atrial fibrillation (%)	8.4	10.1	8.2	0.462
HCM (%)	1.7	3.1	3.5	0.112
Anti-platelet (%)	49.0	53.1	55.3	0.060
Statins (%)	59.1	68.2	65.7	0.057
ACEI (%)	22.0	22.0	22.1	0.514
ARB (%)	49.7	45.3	46.6	0.249
Beta-blocker (%)	59.4	62.9	69.5	0.004
Alpha-blocker (%)	6.6	6.3	6.5	0.514
CCB (%)	55.2	58.5	55.9	0.478
Diuretic (%)	16.1	16.7	20.4	0.076
Warfarin (%)	2.0	2.5	2.6	0.512

Denote: *BUN* blood uria nitrogen, *LDL-C* low-density lipoprotein cholesterol, *HDL-C* high-density lipoprotein cholesterol, *CHD* coronary heart disease, *HCM* hypertrophy cardiomyopahty, *ACEI* angiotensin converting enzyme inhibitor, *ARB* angiotensin receptor blocker, *CCB* calcium channel blocker

### Comparison between male and female OSA patients

Among 685 OSA patients, males were predominant (79% (537 cases)). Compared to male OSA patients (Table 3), females were older, had higher plasma levels of HbA1c, FPG and TC, and higher likelihood of diabetes mellitus and dyslipidemia (*P* < 0.05). Nonetheless, male OSA patients had higher values of AHI, IVS, LVPW and creatinine (*P* < 0.05). In addition, aortic dissection and aneurysm were also significantly higher in male OSA subjects (*P* < 0.05). Notably, except for CHD, cerebrovascular disease, heart failure, atrial fibrillation and HCM tended to be more frequent in females though without statistical significance. No significant differences in medications and other clinical and laboratory variables were observed between the male and female OSA patients.

**Table 3:** Comparisons between male and female OSA patients

Variables	Female	Male	*P* value
N (%)	148 (21.6)	537 (78.4)*	<0.001
Age (years)	62.8 ± 10.2	58.4 ± 11.9*	<0.001
BMI (Kg/m^2^)	26.2 ± 4.2	25.6 ± 4.1	0.136
SBP (mm Hg)	139.8 ± 21.4	136.7 ± 21.5	0.111
DBP (mm Hg)	79.1 ± 15.1	79.4 ± 14.1	0.831
AHI	18.0 ± 15.4	23.1 ± 16.8*	0.001
IVS (mm)	10.7 ± 1.8	11.6 ± 2.4*	<0.001
LVPW (mm)	9.9 ± 1.6	10.7 ± 1.8*	<0.001
HbA1c (%)	6.6 ± 1.4	6.2 ± 1.1*	0.001
Creatinine (μmol/L)	81.7 ± 38.7	103.7 ± 52.2*	<0.001
BUN (mmol/L)	5.7 ± 3.5	5.8 ± 2.6	0.836
FPG (mmol/L)	7.0 ± 3.4	6.3 ± 2.8*	0.015
Triglyceride (mmol/L)	1.8 ± 0.2	1.8 ± 0.3	0.619
Total cholesterol (mmol/L)	4.7 ± 1.4	4.5 ± 1.2*	0.019
LDL-C (mmol/L)	2.8 ± 1.1	2.7 ± 0.9	0.092
HDL-C (mmol/L)	1.1 ± 0.3	1.0 ± 0.2	0.947
Proteinuria (%)	14.9	21.9	0.289
Diabetes mellitus (%)	33.8	21.8*	0.003
Dyslipidemia (%)	23.0	12.3*	0.001
Aortic dissection (%)	10.8	22.3*	0.002
Aneurysm (%)	1.4	6.5*	0.014
CHD (%)	37.8	43.0	0.258
Cerebrovascular disease (%)	12.8	9.1	0.181
Heart failure (%)	25.0	21.0	0.303
Atrial fibrillation (%)	12.8	8.0	0.070
HCM (%)	4.1	3.2	0.595
Anti-platelet (%)	55.4	54.0	0.762
Statins (%)	68.2	66.5	0.687
ACEI (%)	19.6	22.7	0.417
ARB (%)	43.2	46.7	0.450
Beta-blocker (%)	60.8	68.0	0.102
Alpha-blocker (%)	3.4	7.3	0.088
CCB (%)	60.8	56.1	0.300
Diuretic (%)	18.9	18.6	0.935
Warfarin (%)	10.1	6.3	0.112

Denote: *BUN* blood uria nitrogen, *LDL-C* low-density lipoprotein cholesterol, *HDL-C* high-density lipoprotein cholesterol, *CHD* coronary heart disease, *HCM* hypertrophy cardiomyopahty, *ACEI* angiotensin converting enzyme inhibitor, *ARB* angiotensin receptor blocker, *CCB* calcium channel blocker

*P < 0.05 versus female group

### Univariate and multivariate regression analyses

The 971 participants were separated into two groups, namely with OSA and without OSA. In the univariate regression analysis, as shown in Table 4, age, BMI, HbA1c, IVS, IVPW, statins, beta-blockers and uric acid were positively associated with OSA, whereas HDL-C was negatively associated with OSA. We further evaluated the gender-specific differences in risk factors associated with OSA. In the male subjects, as shown in Table 5, after adjusting for potential covariates, only BMI remained positively correlated with OSA (odds ratio (OR): 1.064, 95% confidence interval (CI): 1.008–1.123, *P* = 0.024). In the female subjects (Table 6), after adjusting for potential covariates, only age was still positively correlated with OSA (OR: 1.071, 95% CI: 1.029–1.116, *P* = 0.001).

**Table 4:** Univariate regression analysis

Variables	Odds ratio	95% confidence interval	*P* value
Age	1.019	1.008–1.031	0.001
BMI	1.080	1.039–1.123	<0.001
HbA1c	1.174	1.015–1.358	0.031
HDL-C	0.398	0.232–0.682	0.001
IVS	1.181	1.008–1.283	<0.001
LVPW	1.121	1.032–1.219	0.007
Statins	1.397	1.051–1.856	0.021
Beta-blocker	1.350	1.016–1.794	0.039

**Table 5:** Multivariate regression analyses in male OSA subjects

Variables	Odds ratio	95% confidence interval	*P* value
Model 1			
Age	1.022	1.006–1.073	0.006
BMI	1.081	1.031–1.133	0.001
Model 2			
Age	1.019	1.001–1.038	0.035
BMI	1.066	1.01–1.125	0.02
Model 3			
BMI	1.064	1.008–1.123	0.024

Model 1 was adjusted to SBP and DBP

Model 2 was adjusted to SBP, DBP, DM, Dyslipidemia, FBG, HbA1c, LDL-C and HDL-C

Model 3 was adjusted to SBP, DBP, DM, Dyslipidemia, FBG, HbA1c, LDL-C, HDL-C, Statins, Diuretics and Beta-blocker

**Table 6:** Multivariate regression analyses in female OSA subjects

Variables	Odds ratio	95% confidence interval	*P* value
Model 1			
Age	1.069	1.034–1.105	<0.001
BMI	1.124	1.039–1.217	0.004
Model 2			
Age	1.067	1.028–1.107	0.001
Model 3			
Age	1.071	1.029–1.116	0.001

Model 1 was adjusted to SBP and DBP

Model 2 was adjusted to SBP, DBP, DM, Dyslipidemia, FBG, HbA1c, LDL-C and HDL-C

Model 3 was adjusted to SBP, DBP, DM, Dyslipidemia, FBG, HbA1c, LDL-C, HDL-C, Statins, Diuretics and Beta-blocker

## Discussion

In our cross-sectional study of randomly selected hypertensive patients, the prevalence of OSA was 70.5%, with a predilection for males versus females (78.4% versus 21.6%). Compared to those without OSA, subjects with OSA had higher cardiovascular risk profiles, with the highest risk in those with more severe OSA. Age, BMI, HbA1c, IVS, LVPW, statins, and beta-blockers were positively associated with OSA while HDL-C was negatively associated with OSA in univariate regression analysis. After multivariate regression analyses, with extensively adjusting for potential covariates, only BMI remained significantly associated with OSA in male subjects, while in females, only age remained significantly associated with OSA. Thus there are significant and important differences in epidemiological characteristics between Chinese male and female OSA patients.

Recent epidemiological studies reveal a high prevalence of OSA in the general population [11–13]. OSA is a major public health problem worldwide owing to its close relationship with a variety of CVD such as hypertension [14], diabetes mellitus [15], coronary heart disease [16], cerebrovascular disease [17], atrial fibrillation [18] and congestive heart failure [19]. Mechanistically, subjects with OSA suffer continuous sympathetic nerve and renin-angiotensin axis activation, oxidative stress and systemic inflammation, and all of these pathological alterations are detrimental to cardiac and vascular systems [4, 20, 21]. Results from our present study further confirmed previous findings. In our study, compared to hypertensive patients without OSA, those with OSA were older and had higher values of BMI, cardiac structural changes, and HbA1c (*P* < 0.05 for all comparisons). Furthermore, the incidences of co-morbidities including proteinuria, diabetes mellitus, dyslipidemia, aneurysm, coronary heart disease, cerebrovascular disease, heart failure, atrial fibrillation and hypertrophy cardiomyopathy were also marginally higher in OSA patients than those without OSA, although without statistical significance. All these findings strongly indicated that hypertensive patients with OSA are at increased cardiovascular risk profiles than their counterparts without OSA. The higher percentage of statin and beta-blockers usage in OSA patients might be due to much more morbidities in these populations which required corresponding drugs treatment. Further evaluation and comparison between different degrees of OSA groups showed that there was a dose-effect relationship between OSA and cardiovascular risk (as shown in Table 2) which was consistent with previous reports [13, 22, 23]. Collectively, these findings strongly indicated that hypertension and OSA might confer addictive effects on cardiovascular system and OSA increased risk of CVD in patients with hypertension.

Compared to females, males were reportedly prone to develop OSA owing to gender-specific differences in upper-airway anatomy and function, fat distribution, hormonal status and ventilatory control [24–26]. Indeed, in our present cross-sectional study, we also observed that in randomly selected hypertensive populations, the percentage of male with OSA was nearly 3-folds higher than females (78.4% versus 21.6%, *P* < 0.001), which was similar to previous reports [27, 28]. With regard to traditional risk factors for OSA, no significant differences in systolic and diastolic blood pressure, BMI, FPG and lipid profiles were observed between male and female OSA patients. In contrast, female subjects were significantly older than the males (62.8 ± 10.2 years versus 58.4 ± 11.9 years, *P* < 0.001). With respect to a higher percentage of male subjects with OSA, we considered that male subjects in present study might have a higher prevalence of undetected major risk factors for OSA such as smoking, alcohol abuse and large neck girth, and our ongoing study will include these risk factors so as to demonstrate our hypothesis. In addition, relative small sample size might not be powerful to differentiate these differences, and further studies are warranted to clarify our postulates. Of note, the rates of proteinuria, aortic dissection, aneurysm and coronary heart disease were slightly higher in males, and the rates of diabetes mellitus, dyslipidemia, cerebrovascular disease, heart failure and atrial fibrillation were marginally higher in females, suggesting that there were somewhat differences in epidemiological characteristics between male and female OSA patients.

After extensively adjusting for potential covariates, only BMI remained significantly associated with OSA in males, and age remained an independent contributor to OSA in females. These gender-specific differences in independent risk factors associated with OSA suggest that weight control in males, and modifying age-related pathophysiological alterations such as estrogen and progesterone depletion in females, conceivably may be relevant to mitigating OSA in Chinese hypertensive patients.

Our study has several limitations. First our results can only be used for hypothesis–generation, owing to the inherent limitations of cross-sectional research. Second, the relative small sample size might not be adequately powered to identify other significant differences in characteristics between male and female OSA patients. Third, some important risk factors for OSA, including smoking status, alcohol consumption, neck girth and hormone status, were not evaluated in the present study. Last, we recorded only one blood pressure during admission. Twenty-four hour ambulatory blood pressure monitoring may help better understand the additive effects of OSA and hypertension patterns on cardiovascular systems.

## Conclusion

In our Chinese subjects, the likelihood of OSA in randomly selected hypertensive patients is strikingly and unexpectedly high, especially in males. Hypertensive subjects with OSA have more cardiovascular risk factors than those without OSA. Important differences are evident in male and female OSA patients. Large cohorts and interventional studies are warranted to investigate effective and efficient diagnostic and therapeutic strategies for hypertensive patients with OSA.
